# Influence of Methomyl and Salinity on the Freshwater Snail *Physa venustula*: Egestion, Movement, and Hydration Index

**DOI:** 10.21315/tlsr2025.36.3.1

**Published:** 2025-10-31

**Authors:** Daniel Elias, Jose Iannacone, Jason Doll, Janina Coripuna, Isabel Gonzalez, Alejandra I Herrera, Salvador Inzua, Isbeth I Luyo, Kimberly M Ñahuin, Jaime M Saavedra, Genesis Y Salazar, Giacomo Velasco, Daniela C Vilchez, Rodrigo A Villano

**Affiliations:** 1 Environmental Science Program, North Carolina Wesleyan University, 3400 N Wesleyan Blvd, Rocky Mount, NC 27804, USA; 2 Universidad Científica del Sur, Antigua Panamericana Sur 19, Villa EL Salvador 15067, Peru; 3 Francis Marion University, 4822 E Palmetto St, Florence, SC 29506, USA

**Keywords:** Methomyl, Salinity, Movement, Egestion, *Physa venustula*

## Abstract

Human activities, particularly agriculture and urbanisation often have detrimental effects on aquatic ecosystems and their ecosystem services to varying degrees. Organic pollutants (e.g., pesticides, pharmaceuticals, Per- and Polyfluoroalkyl Substances [PFAS]) and abiotic stressors (e.g., salinity, temperature, pH) are common stressors of freshwater habitats with expanding platforms documenting these problems or issues. This research addresses the effects of individual and combined methomyl (insecticide) and elevated salinity on movement, hydration and egestion of the snail *Physa venustula*. Snails were exposed to treatment concentrations of 100 μg/L of methomyl and 5 g/L salinity for 96 h. Results indicated a significant reduction in snail movement when exposed to salinity (74%), and in combination with methomyl (67%). In contrast, we did not observe significant effects on egestion or hydration across treatments. These findings suggest that there is an energy trade-off to maintain homeostasis from the other physiological processes. Reduced movement can alter feeding rates, predator avoidance behaviour leading to changes in ecosystem structure and function. This research can provide critical insight into how short-term exposure to multiple stressors affects freshwater invertebrates and suggest *P. venustula* may be useful for early detection of water quality changes, especially in regions where chemical monitoring is limited.

**HIGHLIGHTS**

- Elevated salinity (5 g/L) significantly reduced *Physa venustula* movement by 74% compared to the control.
- Combined methomyl (100 μg/L) and salinity exposure reduced movement by 67.
- Methomyl alone did not produce significant effects on movement, hydration or egestion.

## INTRODUCTION

Human activities including agriculture and urbanisation have the potential to affect freshwater environments and their available ecosystems services like drinking water, food and recreation (Kanianska 2016). Elevated salinity in lotic and lentic ecosystems can result from agricultural practices and road salts which change the balance of ions in water (Meybeck & Helmer 1989; Cañedo-Argüelles *et al*. 2019; Hauton 2016). Further, because of climate change and rising sea levels, saltwater moves into groundwater and freshwater ecosystems stressing aquatic organisms by disrupting their osmotic balance (Velasco *et al*. 2019). In these conditions, energy to maintain homeostasis is prioritised from other physiological functions (e.g., growth, movement, reproduction, egestion) (Sokolova *et al*. 2012) affecting ecosystem functions such as food webs and nutrient cycles (Velasco *et al*. 2019).

While salinity alone can affect aquatic organisms, it can also change pollutants toxicity (Meybeck & Helmer 1989). For example, salinity increases the toxicity of aldicarb (insecticide) and cadmium (heavy metal) by increasing their bioavailability on rainbow trout (*Oncorhynchus mykiss*) (El-Alfy *et al*. 2001). PFOS (Perfluorooctane sulfonate) reduced the hatching rate of medaka embryos (*Oryzias melastigma*) with increased salinity (Ye *et al*. 2014). However, lower toxicity of pollutants under elevated salinity has been reported, copper availability decreased with increased salinity (Hall *et al*. 2008) and seleno-L-methionine had a decreased effect on juvenile trout (Schlenk *et al*. 2003) in hypersaline conditions. These findings highlight the complex interactions between different organisms, salinity and other pollutants (Xing *et al*. 2022).

On a larger scale, changes in toxicity can alter population dynamics by affecting survival, growth and reproduction. These shifts can lead to changes in species abundance, community composition and prey-predator relationships. For sensitive species, reduced population growth or altered behaviour can disrupt trophic interactions, grazing pressure, detritus breakdown, nutrient cycling and overall water quality. These disruptions often affect important ecosystem functions such as organic matter breakdown, energy movement efficiency across trophic levels and rates of primary productivity. This highlights the importance of assessing multiple stressors in ecotoxicological research. Methomyl (S-methyl N-[(methylcarbamoyl)oxy]thioacetimidate) is a carbamate insecticide commonly used in agriculture to control aphids (Aphididae), beetles (e.g., Chrysomelidae, Coccinellidae), thrips (Thripidae) and mites (Tetranychidae) on vegetables such as lettuce (*Lactuca sativa*) andonions (*Allium cepa*), fruits including apples (*Malus domestica*) and citrus (*Citrus* spp.), and field crops such as corn (*Zea mays*) and soybean (*Glycine max*) (Lewis *et al*. 2016). Methomyl mode of action is by inhibiting the enzyme acetylcholinesterase resulting on disruption of the nervous system leading to paralysis and death (U.S. Environmental Protection Agency 2018). Because of its high solubility (57.9 g/L) and low soil adsorption (log K_ow_: 1.24), methomyl has the potential to move into surface and groundwater via runoff and leaching (Van Scoy *et al*. 2013) where it has been detected at concentrations of 55.3 μg/L in streams and 400 μg/L in groundwater (Gilliom *et al*. 2006). Thus, given methomyl environmental mobility, its impact may be significant on aquatic organisms.

*Physa venustula* (A. Gould, 1847) is a common snail in South America (WoRMS Editorial Board 2016; GBIF Secretariat 2023) found in the five hydrological basins, including the Colombia-Ecuador Pacific Coast, Magdalena, North Chile Pacific Coast basin, Orinoco and Peru Pacific Coast (Sartini *et al*. 2024). *P. venustula* is a primary consumer feeding on algae, biofilm and detritus (WoRMS Editorial Board 2016) and preyed on by insects (e.g., odonates and hemipterans), crayfish (Orconectes spp.), amphibian and fish (Brown & DeVries 1985; Martin *et al*. 1992). While there is limited peer-reviewed literature on *P. venustula*, research on closely related species such as Physa acuta is abundant and provides insights into their predation dynamics, ecotoxicological responses, physiological functions and metabolic processes.

Physid snails have been used in ecotoxicological research because of their sensitivity to environmental changes and as species indicator of ecological health (Iannacone & Alvariño 1999; Martin 2001; Musee *et al*. 2010; Spyra *et al*. 2019; Henry *et al*. 2022; Kumari *et al*. 2023; Tejada-Meza *et al*. 2023; Wilder *et al*. 2024). Specifically, *P. venustula* movement, egestion and hydration index can serve as proxies of a functioning ecosystem. Changes in movement can affect feeding, predator avoidance behaviour and reproduction (Zukowski & Walker 2009; Wethington *et al*. 2018). Egestion (waste elimination) is critical for growth and it is linked to ingestion and metabolic processes including detoxification (Kumari *et al*. 2023). Hydration index reflects the organisms internal water balance and cellular function (Schmidt-Nielsen 1997).

Our study explores the effects of methomyl and elevated salinity alone and combined on movement, egestion and hydration index of *P. venustula*. We hypothesise:

1. Methomyl will reduce movement, egestion and hydration of *P. venustula* as a result of neurotoxic effects (i.e., inhibition of acetylcholinesterase).
2. We expect similar results from elevated salinity due to changes in osmotic balance.
3. The effect of methomyl and salinity together will amplify the individual effects of these stressors for movement, egestion and hydration index.

Overall, understanding the effects of these contaminants will help predict how aquatic organisms respond to multiple stressors and aid stakeholders on risk assessment and management strategies to protect freshwater ecosystems.

## MATERIALS AND METHODS

*Physa venustula* snails were collected from a pond on the campus of Universidad Científica del Sur, Villa El Salvador, Peru (12°13’12.5”S, 76°58’42.9”W). The pond is located between classroom buildings and the campus cafeteria, surrounded by turfgrass and Ponciana trees (*Delonix regia*). Pesticide application in this area is infrequent and limited, with no documented use of methomyl. To our knowledge, the site is not impacted by agricultural runoff and methomyl contamination has not been reported. Sediment along the pond’s edge was disturbed and snails were manually collected from both the surface and substrate. Collected snails were immediately transferred into sterile containers filled with pond water to minimise stress during transport to the laboratory. Methomyl (Lannate 40SP, Farmex, Lima, Peru) and sodium chloride (100 g, Mandarin Pharmaceutical, Lima, Peru) were purchased to prepare stock solutions.

The bioassay was conducted using 250 mL transparent plastic containers, each containing 200 mL of bottled water (Cielo®) and treatment solution. Water physicochemical parameters were 8.5 pH, 322 mg/L total dissolved solids (TDS), 608 μS/cm conductivity and 34 mg/L chlorides. To reduce evaporation and prevent snail escape, each container was covered with aluminum foil with multiple perforations. The bioassay conditions included a 16:8 light-dark photoperiod and were kept in a greenhouse with temperatures ranging from 16°C to 22°C. Fish food (Nutrafin®) was provided during water changes and treatment renewals.

Four treatments were prepared with 16 replicates each with three snails for each experimental unit (*n* = 64; snails = 192) and maintained over a 96-hour period. We used a 96-hour exposure period to assess early behavioural and physiological responses, similar to other short-term ecotoxicology studies on aquatic invertebrates (Tejada-Meza *et al*. 2023; Fulton & Key 2001; Justice & Bernot 2014; Li *et al*. 2008).

Aliquots of the stock solutions were added to their corresponding treatments to achieve the following concentrations: control (Control, only water), methomyl (Met, 100 μg/L), elevated salinity (Sal, 5 g/L), and combined methomyl and elevated salinity (Met × Sal; 100 μg/L × 5 g/L). The selected treatment concentrations reflect environmentally relevant levels. Methomyl has been detected at 55 μg/L in surface waters (California, US) and 400 μg/L in groundwater (Gilliom *et al*. 2006). Salinity concentration represents levels found in contaminated freshwater ecosystems due to saltwater intrusion (Stockwell *et al*. 2011). Mean snail shell length was 5.25 mm (SD: 0.13 mm), which is consistent with the size range reported for natural populations of *P. venustula* (Martin 2001).

### Movement Assessment

Snail movement was assessed by placing an individual snail on a Petri dish with a 1 cm × 1 cm grid sheet beneath. Each snail was placed at the center of a grid cell with a small amount of water and given a 10-second acclimation period. Movement was recorded for one minute, with each grid cell crossed counted as one unit of movement.

### Egestion Measurement

Egestion rate was calculated based on fecal weight relative to the combined weight of three snails after 96 h (mg/mg/h) relative to the snails’ dry weight. Wet feces were initially weighed, dried in an oven for 24 h at 60°C, and reweighed to obtain the dry fecal weight for analysis. Snail waste was quantified using an analytical balance (Ohaus PR224/E, Ohaus Corporation).

### Hydration Index

Each snail was weighed (wet biomass), wrapped in aluminum foil, and dried in a drying oven (Binder FD 115 E2, Binder GmbH ) for 24 h at 60°C. Soft tissue was not separated from the shell; snails were dried whole to calculate total dry biomass. Post-drying, snails were reweighed to obtain dry biomass data. Snail weight was quantified using an analytical scale (Ohaus PR224/E, Ohaus Corporation). Hydration index was calculated as the percentage difference between wet snail weight and dry snail weight.

### Data Analysis

Movement and egestion rate were analysed using a Kruskal-Wallis test to determine the effect of the four treatments (Control, Met, Sal and Met × Sal) because they were not normally distributed (*p*-values ranged from < 0.001 to 0.090 across groups). Pair-wise comparisons were made using Dunn’s test when an overall statistically significant effect was found. The Kruskal-Wallis test was conducted using the base R package version 4.4.1 (R Core Team 2024) and Dunn’s test was conducted using the FSA package version 0.9.5 (Ogle *et al*. 2023). Hydration index is a percentage and thus modelled using a beta regression with the betareg package version 3.2-1 (Cribari-Neto & Zeileis 2010). Overall effect of treatment variables on hydration index was evaluated using the joint_tests function in the emmeans package version 1.10.5 (Lenth *et al*. 2024). Alpha was set at 0.05 for all statistical tests.

## RESULTS

**FIGURE 1:**
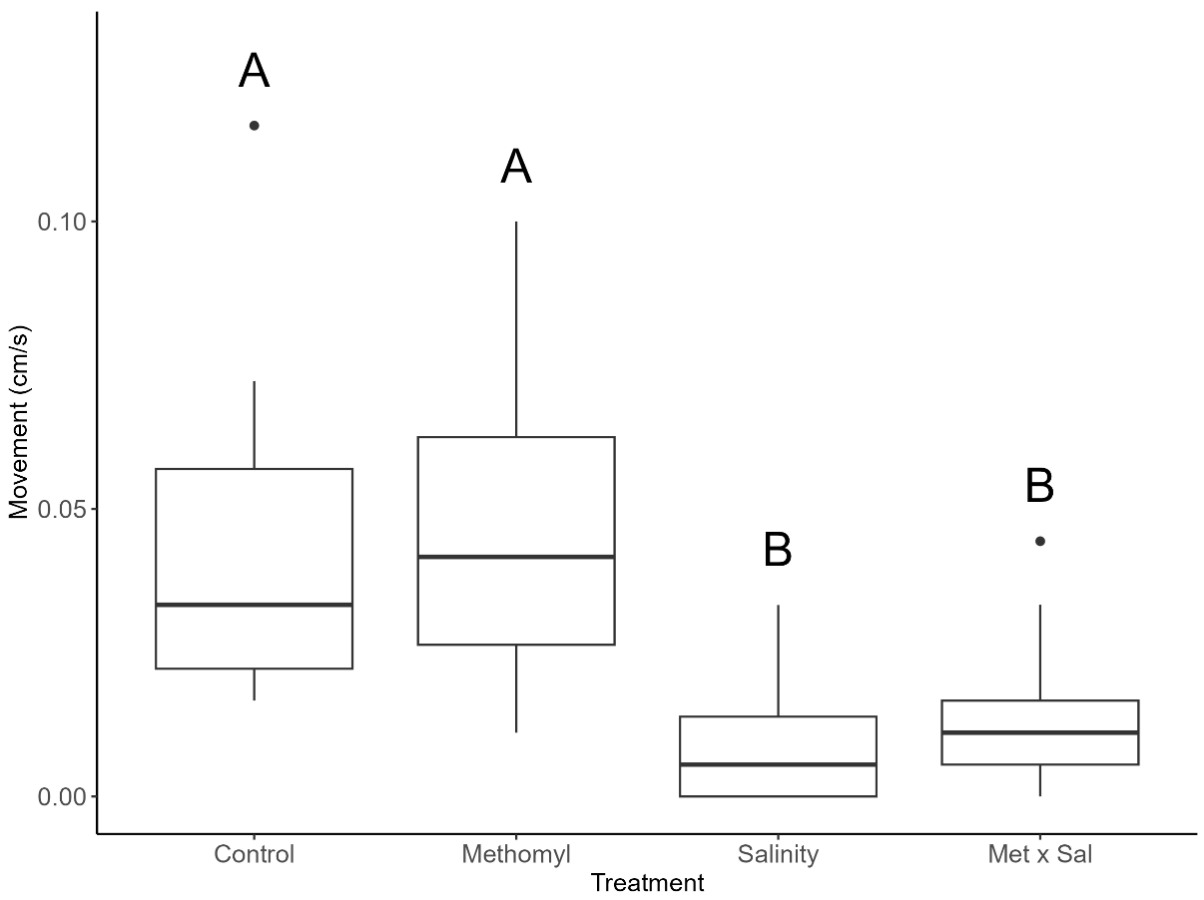
Effects of treatments on snail movement. *Physa venustula* movement (cm/s) as a result of exposure to control, methomyl (Met; 100 μg/L), salinity (Sal; 5 g/L), and methomyl and salinity (Met × Sal; 100 μg/L × 5 g/L). Different letters represent significant differences between treatments.

The mean movement for the control treatment was 0.043 cm/s. For methomyl, we observed an increase of ~7% or 0.046 cm/s. Movement was significantly different across treatments (*X*^2^ = 31, df = 3, *p* < 0.001, Table 1, Fig. 1). Salinity significantly reduced movement to 0.011 cm/s or 74% (*p* < 0.001) relative to the control. Combined Met × Sal significantly decreased snail movement to 0.014 cm/s or 67% compared to the control (*p* = 0.0010). Methomyl alone was not significantly different from the control (*p* = 0.89). Similarly, there were no statistically significant differences between salinity alone and Met × Sal (*p* = 1.0).

**FIGURE 2:**
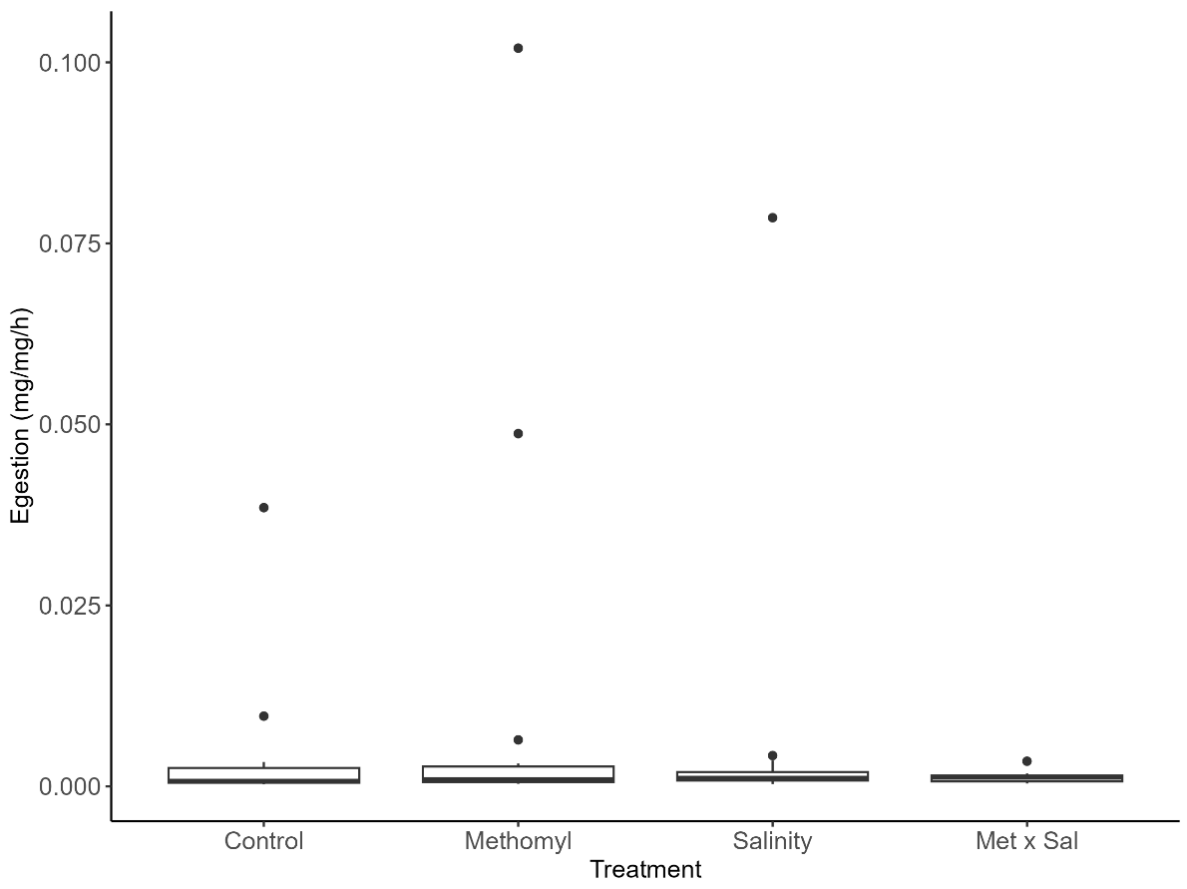
Effects of treatments on snail egestion. *Physa venustula* egestion rate (mg/mg/h) as result of exposure to control, methomyl (Met; 100 μg/L), salinity (Sal; 5 g/L), and methomyl and salinity (Met × Sal; 100 μg/L × 5 g/L). There were no significant differences between treatments.

Egestion rates were not significantly different across treatments (X^2^ = 1.0, df = 3, *p* = 0.79, Table 1, Fig. 2). Methomyl increased egestion rates to 0.011 mg/mg/h or 75% relative to the control (0.0040 mg/mg/h). Salinity exposure resulted in an increase to 0.0066 mg/mg/h or 65% higher than control, while Met × Sal treatment reduced egestion to 0.0012 mg/mg/h or 70% lower than control.

**FIGURE 3:**
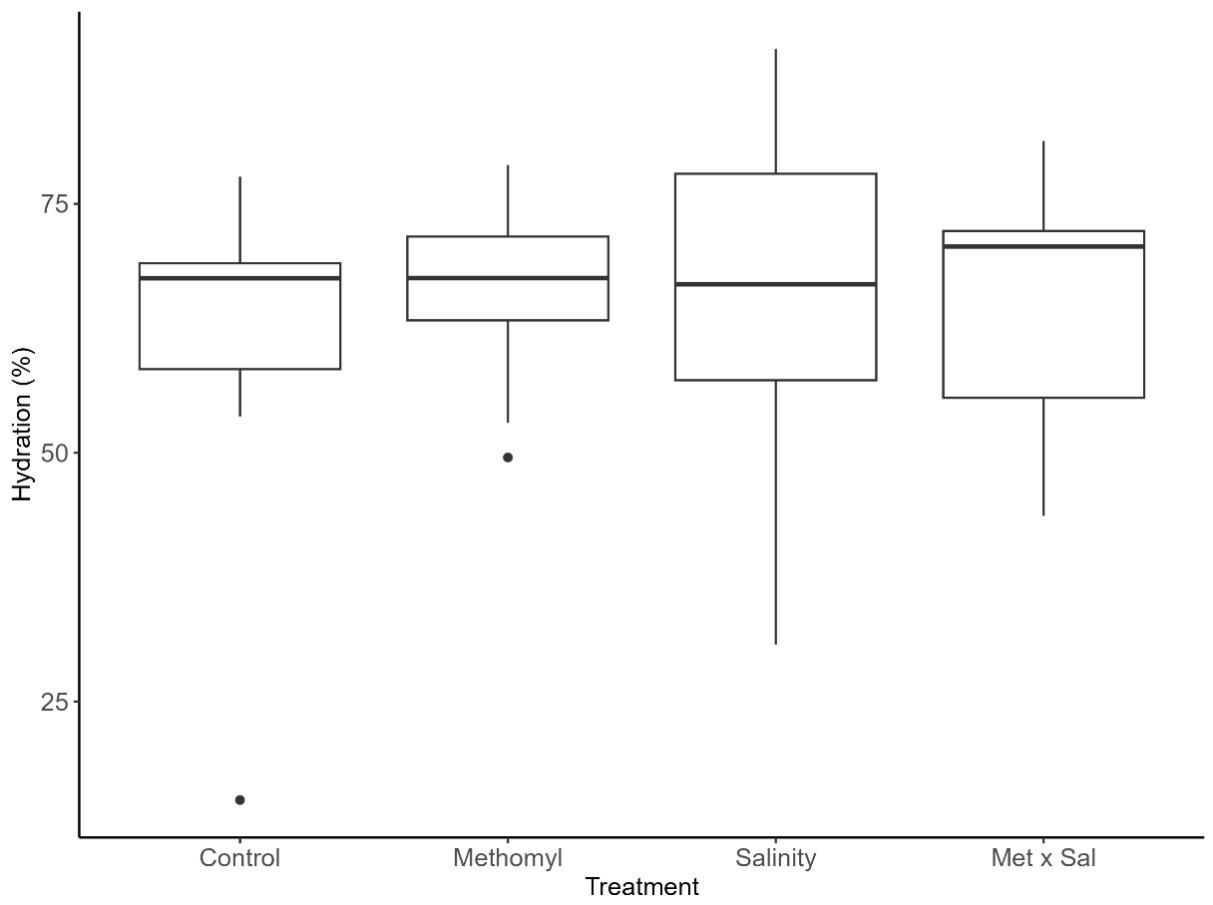
Effects of treatments on snail hydration index. *Physa venustula* hydration index (%) as result of exposure to control, methomyl (Met; 100 μg/L), salinity (Sal; 5 g/L) and methomyl and salinity (Met × Sal; 100 μg/L × 5 g/L). There were no significant differences between treatments.

Hydration indexes were not significantly different across treatments (*X*^2^ = 1.1, df = 3, p = 0.78, Table 1, Fig. 3). Control had a hydration index of 63%, with minimal variation across treatments, with 66% for methomyl and 65% for salinity and Met × Sal treatments.

## DISCUSSION

Our findings showed that elevated salinity alone and in combination with methomyl significantly reduced *Physa venustula* movement. These results indicate that elevated salinity alters osmotic balance of *P. venustula* by changing energy allocation from movement to ionic regulation to maintain homeostasis (Zalizniak *et al*. 2009; Hauton 2016). While our study was not designed to assess synergistic, antagonistic or additive interactions, the reduced movement observed with combined methomyl and elevated salinity exposure appears to be mostly driven by salinity alone, specifically because methomyl by itself had no effect.

Previous studies have shown that insecticides with an inhibitory effect on acetylcholinesterase activity (e.g., organophosphorates, carbamates, synthetic pyrethroids) have a significant effect on motion (Fulton & Key 2001). For example, red drum (*Sciaenops ocellatus*) exposed to azinphosmethyl (6 h, 12 μg/L) had reduced swimming stamina (Van Dolah *et al*. 1997). Similarly, zebra fish exposed to imidacloprid at two concentrations (2.79 ppm or 3.72 ppm) from 4 h to 5 days post-fertilisation showed decreased swimming activity (Malhotra *et al*. 2021). Previous research noted reduced movement for the freshwater snail *Helisoma anceps* exposed to carbaryl (100 μg/L) for 24 h (Elias & Bernot 2017). Thus, the effects of methomyl on aquatic organisms’ movement may vary between species, exposure period and concentrations used Van Scoy *et al*. (2013). Although methomyl alone did not significantly reduce *P. venustula* movement, the concentration used in this research (100 μg/L) is consistent with environmentally relevant levels and within the range used in other ecotoxicological studies. Van Scoy *et al*. (2013) reported that methomyl effects vary depending on the species. For example, *Daphnia magna* showed toxicity at 24 μg/L and *Lepomis macrochirus* (bluegill) affected at 1,050 μg/L. Li et al. (2008) also showed that toxicity increases with exposure time in *Pseudorasbora parva* (topmouth budgeon).

According to the U.S. Environmental Protection Agency (2018), modelled peak surface water concentrations following agricultural use range from 30 μg/L to 99 μg/L and field monitoring has detected values as high as 175 μg/L in surface waters near treated sites. Given this, our study’s lack of a strong behavioural response to methomyl alone is not unexpected, particularly for a non-target snail species that may possess physiological tolerance and the wide response of species to this pesticide. The significant reduction in movement under combined methomyl and elevated salinity exposure suggests that methomyl may have contributed to physiological stress only detectable when additional environmental stressor was present. Higher methomyl concentrations or longer exposures may be necessary to observe consistent behavioural effects under single-stressor conditions. In contrast, we did not observe effects on snail hydration index across treatments. The lack of significant effects may be explained by a close relative to *P. venustula, P. acuta*; this snail effective osmoregulatory mechanisms prevent dehydration (Kefford & Nugegoda 2005). Overall, populations of *P. venustula* from brackish environments or areas with fluctuating salinity may exhibit higher tolerance compared to those from consistent freshwater habitats, suggesting potential local adaptation or acclimatisation (Zalizniak *et al*. 2009). Snails used in this study were collected from water with a salinity of 3.1 g/L, which is classified as mesosaline (Hammer 1986). This is higher than most freshwater systems, which typically have salinity levels below 0.5 g/L and may have contributed to greater tolerance to elevated salinity during the experiment. Thus, while movement was affected by elevated salinity, *P. venustula* mechanisms to maintain ionic balance are adequate to support other physiological functions (Cañedo-Argüelles *et al*. 2019). Additionally, shell length did not differ significantly between treatments indicating that size was consistent across groups and unlikely to influence hydration results.

Snails’ egestion rates did not differ significantly when exposed to individual or combined methomyl or salinity. Pereira *et al*. (2009) reported increased egestion as a detoxification mechanism when exposed to organic pollutants. Further, different responses are associated with elevated salinity (Zalizniak *et al*. 2009) increased ingestion (and subsequently increased egestion) to offset energetic cost of osmoregulation (Burton 1983) and impaired feeding and reduced ingestions leading to decreased egestion as a response to physiological stress (Hawkins & Day 1996). All snails were fed during the water change and treatment renewal, so the variation in egestion was likely due to different physiological responses to the stressors and not food limitation. Our study showed variability within each treatment reflected by the high standard deviation (Zar 1999). This indicates inconsistent responses of the snails to these stressors (Forbes & Calow 2002) or inadequate sample size to detect significance (Cohen 1992). Similarly to hydration index, *P. venustula* energy trade off towards homeostasis may have limited an observable effect on egestion (Sokolova & Pörtner 2003). Egestion and hydration index showed high variability, which may indicate limited sensitivity under short-term exposure. Shell length did not significantly differ across treatments (*p* = 0.94), suggesting that variability in hydration index was not due to size differences. To our knowledge, there is no published data on egestion or hydration index for *P. venustula*, but studies on *P. acuta* allow us to make comparisons. Elias and Bernot (2017) reported control egestion rates of ~0.5 mg/g/h when snails were exposed to pesticides (atrazine, metolachlor, carbaryl and chlorothalonil), while Bernot *et al*. (2005) found higher rates of ~2.28 mg/g/h in snails exposed to ionic liquids. Our control egestion rate averaged 0.004 mg/mg/h (4.0 mg/g/h), which falls within this range. These comparisons support our selection of egestion as a physiological endpoint, although longer exposure durations or larger sample sizes may be necessary to detect consistent treatment effects. Overall, reduced snail movement can lead to reduced feeding thus affecting detritus breakdown and nutrient cycling (Thorp & Covich 2009). P. venustula has a key role in food webs as primary consumer; disruption of its feeding behaviour can contribute to algal growth (Dillon 2000). As a prey, changes in movement can alter snail’s predator avoidance (Turner & Montgomery 2003; Justice & Bernot 2014). In these conditions, snails with decreased motion would be more vulnerable to predation which can lead to a decline of snail population (Alexander & Covich 1991). All these disruptions affect predator prey dynamics altering ecosystem structure and function (Covich *et al*. 1999).

Our study focused on a 96-hour exposure. Acute exposure is commonly used to detect early physiological or behavioral responses in aquatic invertebrates. For example, Tejada-Meza *et al*. (2023) used 96-hour exposure to assess the effects of untreated tannery wastewater on *Daphnia magna, Physa venustula* and *Xenopus laevis*. Sarıkaya (2009) investigated the acute toxicity of alpha-cypermethrin on Nile tilapia (*Oreochromis niloticus*) using short-term exposures (24 h, 48 h, 72 h and 96 h). Fulton and Key (2001) applied short-term exposures (< 96 h) to measure acetylcholinesterase inhibition in sheepshead minnows (*Cyprinodon variegatus*) and invertebrates (e.g., Blue crab, *Callinectes sapidus*; Common prawn, *Palaemon serratus*). Justice and Bernot (2014) studied how nano-silver influenced predator avoidance behaviour in the freshwater snail *Physa acuta* using 24-hour exposure. These studies support the relevance of acute testing in detecting short-term stressor effects. However, longer exposures would provide greater insight into endpoints like hydration index and egestion. Because snails play an important role in freshwater ecosystems and face multiple environmental stressors, it is important to look at how longer exposures affect their behavior and physiology. Chronic studies are needed to better understand these long-term impacts.

Our findings suggest that *P. venustula* may serve as a bioindicator for short-term changes in water quality. Because of the behavioural responses observed in *P. venustula*, specifically the reduced movement after 96-hour exposure to elevated salinity and combined elevated salinity and methomyl, managers could use these snails as indicators of pollutant presence in freshwater habitats. Lotic environments can change rapidly due to rainfall or runoff; chemical monitoring may miss short-lived contamination events. However, behavioural changes offer a “biological snapshot” of stressor exposure. Monitoring snail movement could provide low-cost, field-applicable evidence of water quality in systems where chemical sampling is infrequent or delayed. This approach supports proactive management decisions in locations affected by agricultural runoff, saltwater intrusion or combined stressors, as well as provides valuable information to guide stakeholders in developing effective conservation plans, risk assessment and management strategies.

## Figures and Tables

**TABLE 1:** Mean (standard deviation) values for movement, hydration index and egestion of *Physa venustula* after 96-hour exposure to control, methomyl (100 μg/L), salinity (5 g/L) and combined methomyl and salinity treatments.

Treatment	Movement (cm/s)	Hydration index (%)	Egestion (mg/mg/h)
Control	0.043 (0.026)	63 (14)	0.0040 (0.0095)
Methomyl	0.046 (0.027)	66 (8.4)	0.011 (0.027)
Salinity	0.011 (0.012)*	65 (16)	0.0066 (0.020)
Met × Sal	0.014 (0.013)*	65 (12)	0.0012 (0.00075)

Note: Asterisks (*) indicate values significantly different from the control group (*p* < 0.05).
